# Cerebro-Spinal Fever

**Published:** 1907-07-13

**Authors:** F. M. Sandwith

**Affiliations:** Consulting Physician to Kasr-el-Ainy Hospital


### Hospital Clinics.

### CEREBRO-SPINAL FEVER.

By F. M. SANDWITH, M.D., F.R.C.P., Consulting Physician to Kasr-el-Ainy Hospital.

(A Lecture delivered at the Medical Graduates' College.)

When I was invited to give a lecture in this College and to choose my own subject, I naturally tried to think of some question which I had had the opportunity of specially studying abroad, and which at the same time might have more than an academic interest for those whose routine work is confined to this country. I eventually decided to address you upon the question of cerebro-spinal fever, because, though I cannot pretend to any very profound knowledge of that disease, I have had the advantage of seeing several cases in Egypt and of visiting various hospital wards in the United States when the great epidemic was prevalent there two years ago.

Last year I might have hesitated to address you on this subject, because the disease then seemed to be an exotic from which these islands were unusually free, but now that our few sporadic cases have developed into local outbreaks the position is changed, and it behoves us all to have some intimate knowledge of a malady which we may meet with any day in our ordinary work.

I cannot tell you how many people in Scotland have suffered from this disease this year, but I should like to point out that, whereas in the first quarter of 1906 there were no deaths reported from this cause in the eight principal towns, which represent 38 per cent. of the whole population of Scotland, there were 393 deaths during the first three months of 1907. As many as 312 of the deaths occurred in Glasgow (where a few fatal cases have occurred every year since 1903), besides smaller numbers in Edinburgh, Leith, Paisley, Dundee, and Greenock. The number of deaths is gradually rising, for in January there were only 74, in February 140, and in March 179. The number I have quoted, 393, represents an annual death-rate of 88 per 100,000, being by far the highest death-rate from any acute infectious disease in Scotland, the next on the list this year being whooping-cough.

For Ireland I cannot give you similar figures, but I find in the first quarter of last year no mention at all of cerebro-spinal fever in the registrar's reports; but, on the other hand, during the corresponding three months of the present year cerebro-spinal fever has been recorded in 25 districts, a number only exceeded by one acute infectious disease, for it appears that whooping-cough was present in 27 districts. According to Surgeon-Colonel Flinn the annual deaths from cerebro-spinal meningitis since 1881 have varied from 50 to 133.

The detailed analysis of the death registers for England and Wales for the year 1906 is not yet completed, so that I cannot give you any comparison for this portion of the United Kingdom, but we have all noticed in our weekly medical journals paragraphs referring to the existence of the disease. I find, however, that in London there have been five deaths during the first 13 weeks of this year, and that no less than 13 cases were notified betwen March 12th, when cerebro-spinal fever was made a compulsory notifiable disease in London, and March 30th of this year. These figures, taken from the official records, though necessarily very incomplete, are sufficient to show you that the disease is lurking around us in many localities.

### A Note on its History.

It is now about a hundred years since there appeared in certain parts of France and Italy a form of epidemic disease which the medical men of those countries failed to recognise as belonging to the various groups of fevers then known. Shortly after, the disease was noted in Algiers, to which country it had doubtlessly been carried from France, in the United States, Denmark, and elsewhere. When once its distinguishing features had been pointed out, the disease was soon found over a large part of Europe and at a number of new points in Asia, Africa, and South America. In some of these places there developed widespread epidemics (especially in the United States), which were called "sinking typhus" or "spotted fever." Other names which have been given to the disease are "cerebral typhus," "malignant purpuric fever," "petechial fever," and the one by which most of us know it best, "epidemic cerebro-spinal meningitis."

In some countries sporadic cases are still confused with typhus, so that it is a pity to continue the use of the term "spotted fever," especially when we remember that in many cases of cerebro-spinal fever there is no eruption at all.

It is difficult to tell why the British Isles have, until now, been so free from the disease, for in France and Germany there have been many epidemics, especially among soldiers in barracks. Again, in Denmark, Sweden, Russia, Italy, Sicily, and Greece there are a certain number of cases every winter, and occasionally from them epidemics break out. In hot countries, such as India, it is often the prisoners in gaols who are attacked; and in certain prisons there have been many repeated outbreaks, pointing to local place infection. In Africa it seems certain that the fever has often occurred, though it has usually been called typhus. From Egypt, the Soudan, and Nigeria records are known, and it may interest you to be told that the Mahdi, whose troops killed General Gordon at Khartoum, is believed to have died of this disease, though it was called small-pox at the time by his adherents, who were not possessed of any medical knowledge. In South Africa there are many accounts of this trouble in Natal and in Cape Colony. But North America has really been its headquarters, both as regards prevalence and severity of the disease; for several years it has ranged from Canada to the Gulf of Mexico, and from the Atlantic Ocean to the States of Minnesota and Iowa. It has been said that the fever is so often met with in Alaska that it must be reckoned with as one of the dangers of gold mining in that inhospitable region.

### The Epidemic Form.

The epidemics which distinguish this disease from other forms of meningitis vary widely both in their extent and intensity, and in several instances the outbreaks are of so limited a nature, the disease attacking a mere handful of people, that the word "epidemic" is out of place in connection with them. With the exception, perhaps, of Glasgow, these are the outbreaks which are now going on in the United Kingdom.

It has often been noticed that the epidemics are curiously irregular in their relations both to space and time. Instead of sweeping through a country, like influenza, for instance, they rather tend to occur as a number of isolated outbreaks, here and there, in different parts of the country, the separate outbreaks showing little or no relation to each other; and with our present knowledge it is often impossible to trace any connection between them. So, too, an individual outbreak often shows no definite course; a local epidemic may smoulder on for months, and may recur at irregular intervals in the same district or village, showing that the infection is endemic for the time being in that particular spot. This tendency to recur seems to show that the poison of the disease can exist for a short time outside the human body.

Although there cannot be many people of our own generation who have seen many cases of the disease in England until lately, I may remind you that history relates several groups of cases scattered about England, Scotland, and Ireland; for instance, on Dartmoor, in Sunderland, Liverpool, Rochester, Lincolnshire, Oxford, Birmingham, and several villages in Norfolk and Suffolk. Again, there have been many cases in Dublin, Belfast, and other parts of Ireland, and in 1865 and later the epidemic was so fatal and so often accompanied by dark-coloured eruptions that the name of "black death" was at one time proposed for it.

During the last thirty years several cases have been seen in Dundee, Kilmarnock, Aberdeen, and other parts of Scotland.

The concentration of individuals in barracks, schools, and prisons seems to be a special factor in the spread of this fever, and doubtless accounts for its having been so often confused with typhus. Many of the epidemics on the Continent show us how liable recruits and young soldiers are to the disease. In ordinary civil life it is children and young adults up to the age of thirty who are most susceptible; babies in arms do not often suffer, nor do people over fifty years of age. Koplik, of New York, has, however, reported eight fatal cases less than one year of age. Infants of five months have been known to recover. Speaking generally, the older the child, the better are its chances of recovery.

People have often argued that this disease is not contagious, because they have not met with more than one or two cases in a house, for in a crowded city the distribution of the patients is very scattered; and, besides, in a well-ventilated hospital neither nurses, doctors, nor adjoining patients contract the fever from cases under treatment. The arguments in favour of the disease being considered highly infectious are, that in crowded houses you will often find several cases in one family; and there have been instances—perhaps due to faulty ventilation—of both nurses and patients developing the disease from cases in the hospital wards. Perhaps we may compare it to certain outbreaks of pneumonia, which at times in a family or school present a high degree of malignant infection. When I reached Boston, in the United States, in the spring of 1905 I found that the cerebro-spinal patients were carefully isolated in special wards, and their attendants were not allowed to mix with the rest of the hospital staff. The nurses all wore a white overall and covered up their nose and mouth with several folds of gauze bandage. On the other hand, when I went on to New York, and found the hospitals there crowded with similar patients, no great precautions were taken in addition to treating the patients in special wards, because the medical officers had never seen the disease directly communicated to anyone else.

In order to prevent the spread of the disease, it is a good thing to examine bacteriologically the nose and throat of hospital attendants caring for infected patients. The diplococcus is often found in them, and in such cases the nose and throat should be carefully disinfected. But it is not universally allowed that the avenue of infection is through the nose, for in 1905 Flexner, in New York, found that monkeys could be infected without great difficulty with *diplococcus intracellularis* and made to reproduce the pathological conditions present in man in cerebro-spinal fever. His experiments proved that the cocci, when introduced into the spinal canal by lumbar puncture, distributed themselves in a few hours through the meninges and excited an inflammation, the exudate of which accumulated chiefly in the spinal membranes and those at the base of the brain. The uniformity with which the chief exudate was found at the base of the brain, and the rarity of its appearance in large amount over the convexity, led him to doubt whether it was right to attribute this same localisation in man to the entrance into the meninges of the infective agent directly through the nasal mucous membrane. The inflammation of the meninges extends in monkeys into the membranes covering the olfactory lobes, and, Flexner says, into the ethmoid cells and into the nose. In some cases he found the nasal mucous membrane inflamed with small hæmorrhages.

Dr. Hare, of Philadelphia, has reported a striking case, quoted by Professor Osler, which should be brought to the attention of all non-contagionists. A patient came into the town from a region in which cerebro-spinal fever was prevalent, and died within 48 hours. His physician, a very strong, robust man, of about 26 years of age, was a devoted friend of the patient, and sat up with him until he died. Within 24 hours of the death of his patient the doctor was attacked, and died 48 hours after the onset. Dr. Hare, who had seen the original patient, and had attended the medical man, a day or two later had a slight fever with headache and stiffness of the neck; but in his case the attack passed off with great rapidity. There can be no doubt on this occasion that the poison was communicated directly from the patient.

The duration of cerebro-spinal cases varies extremely, from nine hours to several months. The average course of all cases is about 20 days, but the average of fatal cases is much less.

In New York alone, during the year I was there, no fewer than 2,755 cases of cerebro-spinal fever were reported, and probably many mild cases were not notified. Of the number reported, 1,511 died—a mortality of about 55 per cent. The diplococcus is found in the mucus of the nose in at least half the cases during their first week; it is, fortunately, of feeble vitality, being rapidly killed by drying and by exposure to sunlight, which makes the probability of infection by dust very unlikely. Cerebro-spinal fever in other animals has apparently no connection with the disease in man, always excepting the guinea-pigs and monkeys injected in laboratories. No one has yet thrown any light on the variation which causes some epidemics to affect infants, others older children, and others chiefly adults. The incubation period seems to be short, say from one to five days. Cerebro-spinal fever was originally regarded as an entirely epidemic disease, but of late years many have been converted to the view that the type of meningitis called posterior basic is really due to the same organism, the *Diplococcus intracellularis meningitidis*, and that, therefore, the cases of posterior basic meningitis, so well known in the children's hospitals of this country, are really only sporadic forms of the disease we are considering to-day.

One of the chief differences between cerebro-spinal fever and the forms of meningitis due to tubercular disease and the pneumococcus is that in the former the spinal cord is much more involved than in the latter, where the membranes of the brain are chiefly attacked. The clinical consequence of this is that in cerebro-spinal fever we get special symptoms which are rare in the other forms of meningitis—*e.g.*, stiffness of the neck, rigidity of muscles, sensitiveness of the skin, great retraction of the head, and sometimes opisthotonos. In cerebro-spinal fever the number of recoveries varies from 20 to 50 per cent., whereas in meningitis due to the tubercle bacillus, the streptococcus, or the staphylococcus, recovery is so rare that we hardly ever meet with it.

### Some Points in the Symptomatology.

I do not propose to trouble you with a detailed description of the many symptoms of this disease, because you are familiar with them as described in such text-books as that of Professor Osler, who has, in addition, written an important monograph upon the subject for his Cavendish Lecture at the West London Medico-Chirurgical Society. But there are a few points I should like to refer to briefly. One distinct characteristic of the fever is the abrupt onset, even in cases which do not belong to the fulminating type, whereas you know perfectly well that in tubercular meningitis the onset is slow and insidious. Some slight or marked rigidity in the neck is present at one time or another in all cases of cerebro-spinal meningitis, and opisthotonos is present in about 70 per cent. of the children affected.

In the majority of cases the skin and tendon reflexes are present in the early stages, but are apt to disappear before death in the rapidly fatal cases. The Babinski reflex is of little value in children below two years of age, who constantly have it in normal health or when suffering from other diseases; but the presence of this reflex is much more likely to occur in older children when they are suffering from tuberculous meningitis, as opposed to the cerebro-spinal form. You will remember that Babinski described extension of the great toe on irritation of the plantar surface of the foot as a characteristic sign of disease of the pyramidal tracts or of the lateral columns of the cord.

The contraction of the flexor muscles of the lower extremities, which we call Kernig's sign, is again of very little utility in children under two years of age, because the irritability is so great that it may be impossible to come to any conclusion as to its presence or absence; but it is more likely to be present in cerebro-spinal than in tuberculous meningitis. It was found in 115 cases of the former, out of 132. Hyperæsthesia and mental irritability are well marked in cerebro-spinal fever, whereas the child in tuberculous meningitis lies in a stuporous condition, hardly noticing its surroundings. Facial paralysis is a constant feature of the tuberculous form, but only occurs in the severest types of cerebro-spinal meningitis. The fever is so irregular that there cannot be said to be any characteristic curve.

Sometimes no eruption can be discovered, but often there is herpes on the lips, or brownish stains, or a petechial rash, which looks as if a penful of ink had been spluttered over the skin. On examining the blood, you will usually find leucocytosis present, sometimes as high as 40,000 per c.m.

The absolute diagnosis is, of course, formed by the detection of the diplococcus in the serum of the spinal canal; it must not be forgotten that the diplococcus may not be found by lumbar puncture after the first ten days of the illness. Also that the cerebro-spinal fluid will not remain sterile, and becomes full of bacteria in a few hours; hence it must always be used fresh. The normal amount varies greatly, often from 5 to 20 c.c.; the fluid is relatively most abundant in the first years of life. In cerebro-spinal meningitis there may be as much as 100 c.c., and the colour, instead of being absolutely limpid, or with a slight yellowish tinge, due to the pigment of blood serum, is whitish in this disease, looking like water which has been poured into a milk-stained glass. The alkaline reaction and the specific gravity (1,006 to 1,010) remain unchanged. In normal fluid there is found a body which reduces copper but not bismuth, does not ferment, and is optically inactive; this body is absent in the fluid of cerebro-spinal fever. The total solids found in normal fluid are less than 1 per 1,000, but are considerably increased in meningitis, especially if purulent.

The symptoms of a typical and severe form of cerebro-spinal fever are unlikely to escape recognition by medical men if they have some knowledge of the usual symptoms, such as a sudden onset of shivering, intense headache, giddiness, and persistent vomiting, with an early profound disturbance of the nervous system, betokened by delirium, alternating with somnolence or stupor. A painful spasmodic condition of certain muscles, especially the posterior muscles of the neck, causes retraction of the head; and increased hyperæsthesia of the surface of the body, and sometimes petechiæ or mottling of the skin, are commonly noticed.

But when the fever appears in milder or anomalous forms, identification is naturally more difficult, and in this country mistakes have occasionally been made. Some seventeen years ago there were some localised outbreaks in the Eastern counties, which were generally mistaken for sunstroke or for typhoid fever, while in Northamptonshire similar cases were called pneumonia or sore throat; and as late as two years ago there were patients at Irthlingborough, near Northampton, who were thought to be suffering from influenza. Here is another instance of faulty diagnosis: During a small outbreak of the fever in Cairo, a Greek boy, aged six, was admitted to the German hospital, because it was thought by several medical men that his symptoms were due to cerebro-spinal meningitis. Arrangements were accordingly made to confirm the diagnosis by lumbar puncture, but before the operation could be done the nurse reported that the boy had passed an Ascaris lumbricoides. When santonin was administered, 39 similar worms were expelled, and his nervous symptoms immediately disappeared!

### Aids to Diagnosis.

I have so often referred to the confusion of one disease with typhus that I must not omit all mention of it while considering differential diagnosis, especially as typhus has become so rare a disease in England that the rising generation seldom sees it. Typhus is not characterised by the severe pains in the head and back of the neck common in cerebro-spinal fever, the eruption is general, and I have never known herpes to appear. The duration of the two diseases is different, for typhus lasts for two weeks, while this fever is either shorter or longer.

### Lumbar Puncture.

Lumbar puncture is best performed when the patient is lying on his right side, with knees drawn up and the left shoulder turned forward. The needle of an antitoxin syringe is introduced midway between the third and fourth, or the fourth and fifth, lumbar vertebræ, below the spinous process, a little to one side of the median line, the thumb of the left hand of the operator being placed between the spinous processes as a guide. The needle should be directed slightly upwards and inwards, and should enter the canal at the depth of three-quarters of an inch in infants, and from one inch and a half to two inches and a half in adults. The syringe should then be unscrewed, and the fluid allowed to fall drop by drop into a sterilised test-tube, until from one to three drachms have been withdrawn for bacteriological examination. If the puncture is being used with the idea of palliation or cure, of course a greater quantity may be removed.

The most common complication of the disease seems to be otitis in children; adults more often have joint complications. Pleurisy, pericarditis, parotitis, persistent headache, and optic neuritis have all been seen as sequelæ.

### Treatment.

Hot baths for rigors, sponging for fever, and ice to the head or spine have been employed in many epidemics. Various drugs have been given, without any convincing results. In New York I saw cases treated with ergot, with the idea of trying to contract the blood-vessels; children had half a drachm of the fluid extract per rectum twice a day, and adults were given one drachm every three hours. I also saw several dozen patients treated with large doses, up to 30,000 or 40,000 units of diphtheria antitoxin, usually administered intra-muscularly, but sometimes intra-spinally. This empiric treatment was fashionable for a few months on apparently no better grounds than that the diplococcus was found not to grow in the antitoxin. When I asked one of the best-known and best-respected physicians in New York what his then treatment was, he said: "I am now trying acetate of copper by the mouth, for in this disease one clutches at every straw, even if it be not a straw."

In many cases repeated lumbar punctures have helped the fever, headache, delirium, and general irritability of the patient, and there is no rule as to the number of times a case may be punctured, though one generally waits for four or five days before a repetition. When the fluid flows very rapidly from the cannula, as much as three ounces may be removed; but if the fluid flows slowly, drop by drop, one ounce will usually be sufficient to relieve all tension. Sometimes puncture has been followed by hæmorrhage, probably from the veins in the subdural space, but no ill effects have been known to follow.

### The Serum Treatment.

Flexner has made a serum from artificially infected monkeys, which may some day prove to be of prophylactic value. Some cases have lately been treated by vaccine prepared from their own serum. Perhaps the worst form of treatment is that adopted in some parts of the Soudan, where the popular belief is that when a patient is unconscious or delirious he has devils in the head, and the only way to drive out these devils is by collecting as many of the women of the village as possible to congregate around the sick man. Then these women, with the help of the tom-tom and the weirdest shrieking, successfully drown his delirium; but one shudders to think of the effect of the treatment on the intense headache of the unfortunate patient.

